# Cultivating Community: Building Structures of Caring in Gerontological Spaces

**DOI:** 10.1093/geroni/igaf122.437

**Published:** 2025-12-31

**Authors:** Nicholas DiCarlo, Brittney Pond, Díana Poncé

## Abstract

Research suggests that building community is important for mental health, personal and professional growth, feelings of belongingness, etc. (Brower, 2020). Scientists, researchers, and academics have made efforts to form community through various avenues, such as early career writing groups (Kent et al., 2017), informal mentorship structuring (Pegg et al., 2015), and more. In this symposium, we aim to explore two interconnecting questions: 1) how do we create structures of caring within communities during times of crisis (such as the COVID-19 pandemic, sociopolitical change, etc.) 2) what is the role of resistance in healing? The three presenters address this question using various methods, including community reflections, personal experiences, and data-driven discussions. This symposium has several practical and theoretical implications, such as 1) strategies for navigating disruptions 2) ways to cultivate community and 3) discussions regarding the meaning of community within and beyond the academy. These contributions are applicable to faculty and staff, researchers, clinicians, students, community members, participants, etc. This symposium aims to include active audience discussion and resource sharing to build community specifically at GSA.

